# Asthma in Young Children: Prenatal DDE Exposure May Increase Risk

**Published:** 2005-12

**Authors:** Kris Freeman

Most countries have banned the agricultural use of the organochlorine insecticide DDT because of the way this persistent, fat-soluble compound accumulates in the food chain. However, DDT is still widely sprayed in developing countries to combat malaria-bearing mosquitoes. Studies have linked exposure to DDT and its persistent metabolite *p,p*′-DDE to changes in the immune responses of human cells, and to asthma prevalence in children and adults. A longitudinal study now shows that prenatal exposure may provide the fundamental window for asthma susceptibility linked to DDT **[*EHP* 113:1787–1790]**.

Investigators collected umbilical cord blood from 482 children born on the Spanish island of Menorca and tested 84% for the presence of organochlorine compounds. DDT is not used on Menorca. However, the parents of the children in the study ate relatively large amounts of fish, which can be a source of exposure to DDT residues. According to self-reports of diet on questionnaires, more than half of the mothers ate fish more than twice a week during pregnancy.

All of the children tested had *p,p*′-DDE in their cord serum (the median concentration was 1.03 nanograms per milliliter [ng/mL]). Serum levels tended to be higher in children with older mothers, although the mothers’ fish consumption during pregnancy correlated poorly with the children’s DDE levels. Each child’s serum also contained hexachlorobenzene and polychlorinated biphenyls.

The researchers correlated the children’s prenatal exposure to risk of having asthma or atopy at age 4. Asthma was defined as one or more episodes of wheezing in the fourth year alone, one or more episodes of wheezing per year in consecutive years (“persistent wheezing”), or a physician’s diagnosis of asthma. Atopy was defined as having blood levels of specific immunoglobulin E antibodies for dust mites, cats, or grasses. Of the initial participants, 97% provided medical information yearly through age 4 and 75% provided blood samples at age 4; 306 of these samples were tested for antibodies and for peripheral white blood cells, a sign of the underlying inflammation responsible for asthma.

Wheezing was reported at age 4 for 11.6% of the children whose blood was tested for organochlorines. In addition, 12.6% of those who gave blood at age 4 had antibodies for the specified allergens in their blood. The risk of wheezing increased with the concentration of *p,p*′-DDE in the child’s cord serum. Of the children in the lowest quartile of exposure (less than 0.57 ng/mL), 9% reported wheezing compared to 19% of the children in the highest quartile of exposure (more than 1.90 ng/mL). There were no correlations between wheezing in the children and maternal consumption of fish during pregnancy.

There was no apparent link between atopy and the relationship between DDT and wheezing; children both with and without atopy had a similar increase of wheeze with increasing *p,p*′-DDE. The researchers speculate that the lack of an association between DDT exposure and atopy in their study could be due to the young age of the children studied, as sensitization to allergens tends to increase during childhood. There was no correlation between the other organochlorine compounds measured and wheezing or atopy.

Further study is needed to determine if the link between DDT and asthma susceptibility is caused by the effect of the insecticide on the immune system or the hormonal system. In addition to its direct impact on immune cells as shown in previous research, *p,p*′-DDE has also been shown to interfere with hormonal receptors and to mimic estrogen activity, which might indirectly affect immune responses. The researchers suggest that their results be considered when evaluating the risk of spraying DDT in anti-malaria campaigns.

## Figures and Tables

**Figure:**
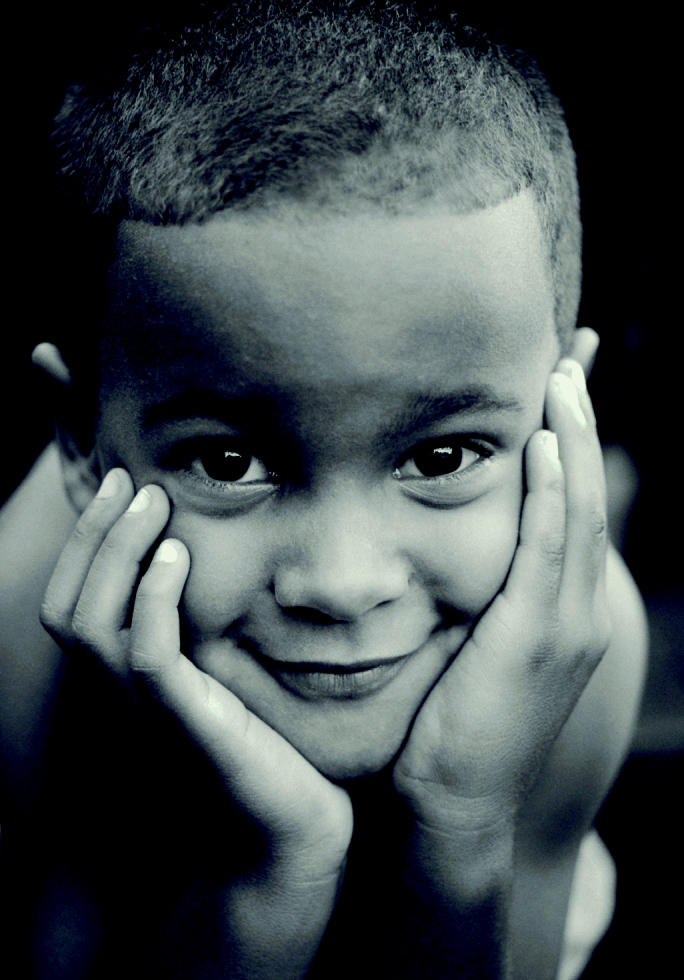
Then and now. A study of Spanish mother–child pairs shows that DDT exposure *in utero* may contribute to later asthma in children.

